# The Concentration of Follistatin and Activin A in Serum and Selected Biochemical Parameters in Women with Polycystic Ovary Syndrome: Stratification by Tobacco Smoke Exposure, Insulin Resistance, and Overweight/Obesity

**DOI:** 10.3390/jcm13175316

**Published:** 2024-09-08

**Authors:** Justyna Niepsuj, Agnieszka Piwowar, Grzegorz Franik, Anna Bizoń

**Affiliations:** 1 Department of Toxicology, Faculty of Pharmacy, Wroclaw Medical University, 50-556 Wroclaw, Poland; justyna.niepsuj@student.umw.edu.pl (J.N.); agnieszka.piwowar@umw.edu.pl (A.P.); 2 Department of Endocrinological Gynecology, Medical University of Silesia, 40-752 Katowice, Poland; gfranik@sum.edu.pl

**Keywords:** polycystic ovary syndrome, tobacco smoke exposure, overweight/obesity, follistatin and activin A

## Abstract

**Background/Objectives**: The aim of the study was to investigate the concentrations of follistatin and activin A in the serum of women with polycystic ovary syndrome (PCOS) and to assess their relationship with selected biochemical parameters, specifically stratifying the analysis based on tobacco smoke, insulin resistance, and abnormal weight. **Methods**: The research was carried out within a cohort of 88 women (60 women with and 28 without PCOS). **Results**: We observed significant differences (*p* < 0.05) in follistatin concentrations between women with PCOS stratified by homeostatic model assessment for insulin resistance (HOMA-IR) values. These differences were consistent across both smoking and non-smoking subgroups with PCOS. Similar results were observed when comparing normal-weight women with PCOS to those with overweight or obesity. Additionally, activin A concentrations were significantly increased by higher body mass index (BMI) and HOMA-IR values in non-smoking women with PCOS. Moreover, we identified a negative correlation (r = −0.30; *p* < 0.023) between cotinine levels and Anti-Müllerian hormone. Among smoking women with PCOS, we noted decreased concentrations of sex hormone-binding globulin and high-density lipoproteins, alongside increased fasting glucose, insulin, HOMA-IR, and free androgen index values. **Conclusions**: Our findings suggest that activin A and follistatin concentrations are more strongly influenced by disruptions in glucose metabolism and BMI than by tobacco smoke exposure. The observed changes were more pronounced in follistatin than in activin A level.

## 1. Introduction

Polycystic ovary syndrome (PCOS) is a heterogeneous disease with difficulties in estimating etiological factors related to both its development and progression [1]. Many hormonal and metabolic disorders are identified in the course of PCOS [2]. Hyperandrogenism, ovulatory dysfunction, and polycystic ovaries are the main features of this disorder. However, PCOS is also associated with many other disturbances, such as obesity, chronic low-grade inflammation, insulin resistance (IR), type 2 diabetes (T2D), and infertility [3,4]. Many molecules present in the human body play a pivotal role in both metabolic and hormonal status. One of them is follistatin, and the other is activin A, both of which could be crucial parameters during PCOS but have not been sufficiently investigated.

Follistatin is a small, single polypeptide chain glycoprotein that was first discovered in 1987 in porcine ovarian follicular fluid. Its first known function was the inhibition of follicle-stimulating hormone (FSH), which plays an important role in the regulation of female fertility [5]. However, we now know that follistatin has multifunctional activities. The primary function of follistatin is to antagonize transforming growth factor β (TGFβ) superfamily ligands, but it is also involved in muscle growth, reproduction, β-cell function, regulation of insulin sensitivity, and low-grade inflammation [6]. It has been reported that follistatin may hold potential in the treatment of obesity and related diseases, primarily due to its blocking of TGFβ ligands [7]. Additionally, it influences the extent of oxidative stress (OS), which arises from an imbalance between the production and elimination of reactive oxygenated species (ROS) [8]. Given that OS is implicated in the pathogenesis of PCOS [9], it is plausible that follistatin may also impact the course of PCOS by modulating the intensity of OS. Conversely, elevated levels of follistatin have been linked to a heightened risk for T2D and IR [10]. Furthermore, there is evidence indicating a positive correlation between follistatin and cardiovascular risk factors, including lipid levels and markers of adiposity in both women with and without PCOS [11]. 

Activin A is also closely associated with follistatin level and sex hormone in the female body and plays a dual role by promoting follicular development and FSH secretion while inhibiting androgen production in thecal cells [12]. As a member of the TGFβ superfamily, activin A also binds follistatin, which neutralizes its effects. Consequently, an excess of follistatin may lead to impaired follicular development and hyperandrogenism, both crucial characteristics of PCOS [13]. Activin A was also found to regulate multiple aspects of female reproduction. Studies on rodents and humans suggest that it stimulates the differentiation of primordial follicles into antral follicles but can also contribute to follicular atresia. Additionally, activin A modulates the levels of progesterone and human chorionic gonadotropin (HCG) in human placental cultures. Furthermore, studies indicate an imbalance between follistatin and activin A in the blood of women with PCOS compared to controls, suggesting it as a potential causative factor in PCOS [14]. 

Smoking is another crucial factor that significantly impacts female fertility, including ovarian and oviduct function, as well as reproductive hormones [15,16]. Components of smoke affect vascular structure and induce endothelial damage, which can lead to reduced ovarian tissue perfusion [17]. The endocrine effects of nicotine and cigarette smoke have been revealed by other authors [18]. Furthermore, it has been suggested that the management of PCOS should not only include monitoring blood glucose, blood pressure, weight, diet, exercise, sleep, and mental health but also consider exposure to tobacco smoke [19,20].

A study by Tao et al. [21] observed a potential association between initiating smoking and an increased likelihood of developing PCOS. Their findings indicated a likely causal association between smoking initiation and an increased risk of PCOS, highlighting smoking as an important factor in both the etiology and prevention of PCOS. This is particularly significant given the increasing prevalence of PCOS among women and the challenges in identifying its pathogenesis and diagnosis [22]. It was also shown that smoking can inhibit aromatase activity, which is involved in estrogen production [23]. Additionally, decreased Anti -Müllerian hormone (AMH) concentrations [24] and increased androgen levels were observed in the blood of smoking women [25]. 

Based on the results of our previously published studies [26,27,28], where we observed the harmful effect of smoking on female fertility, the aim of the study was to investigate the concentrations of follistatin and activin A in the serum of women with PCOS and to assess their relationship with selected biochemical parameters, specifically stratifying the analysis based on tobacco smoke, insulin resistance, and abnormal weight. 

## 2. Materials and Methods

### 2.1. Materials

The research was carried out within a cohort of 88 women who were admitted to the Gynecological Endocrinology Clinic at Silesian Medical University in Katowice, Poland, during the year 2022. Using the Rotterdam criteria established in 2003 [29], requiring at least 2 of 3 features: ovulatory dysfunction (OD) (amenorrhea or menstrual cycles > 35 days), hyperandrogenism (HA) (biochemical and/or clinical), and polycystic ovarian morphology on ultrasound [30]. A total of 60 women were identified and confirmed to have PCOS, and 37 were non-smoking and 23 were smoking, while 28 non-smoking women were determined not to have PCOS. Furthermore, the Rotterdam criteria allow to determine the 4 phenotypes of PCOS, including phenotype 1 presenting OD, HA, and PCOM; phenotype 2 involving OD and HA; phenotype 3 presenting HA, and PCOM; and phenotype 4 comprising OD and PCOM. The prevalence for each phenotype was for phenotype 1 68.3% (n = 41/60), phenotype 2 6.7% (n = 4/60), phenotype 3 20% (n = 12/60), and phenotype 4 5% (n = 3/60). 

Women in the control group were admitted for the diagnosis of PCOS due to menstrual disorders, but hormonal and imaging tests excluded PCOS as the cause of these abnormalities. Their hormonal profile was normal. The study and control groups excluded individuals who exhibited alcohol abuse. Additionally, individuals diagnosed with hyperprolactinemia, diabetes (type 1 or type 2), hypertension, Cushing’s syndrome, or adrenal tumors were excluded from the study.

Ethical approval for the study was obtained from the Bioethical Committee of the Wroclaw Medical University (No. 222/2024). Blood samples were collected from all participants during the follicular phase of their menstrual cycles (within 1 to 5 days) in the morning, following an overnight fast of more than 8 h, in accordance with established protocols. The collected blood samples were centrifuged, serum or plasma was separated, and stored at −80 °C until assays.

### 2.2. Methods

During hospitalization, anthropometric measurements including height, weight, waist circumference, and hip circumference, as well as the Ferriman-Gallwey scale of hirsutism, were performed.

The exposure to tobacco smoke was evaluated according to a personal survey and the determination of cotinine (Cotinine ELISA, Ref. No. CO096D, Calbiotech Inc., El Cajon, CA, USA), which is the main metabolite of nicotine in biological fluids. In this method, both serum and cotinine enzyme conjugate were introduced to wells coated with anti-cotinine antibodies. Cotinine in serum competed with a cotinine horseradish peroxidase conjugate for binding sites. The addition of 3,3′,5,5′-tetramethylbenzidine resulted in a color intensity, which was measured at λ = 450 nm and found to be inversely proportional to the concentration of serum cotinine. 

The concentration of follistatin was assayed using Human Follistatin Quantikine^®^ (Thermo Fisher Scientific, Waltham, MA, USA) ELISA, Ref. No. DFN00, R&D System, Minneapolis, MN, USA. The methods are based on the quantitative sandwich enzyme immunoassay principle. A microplate is initially coated with a monoclonal antibody that is specific to human follistatin. Samples and standards are introduced into the plate’s wells, where any follistatin present becomes immobilized through binding to the coated antibody. After the removal of any unbound substances by washing, an enzyme-linked monoclonal antibody is added to the wells. After another washing step, a substrate solution was introduced into each well. The color development process was halted, and the absorbance at λ = 450 nm was measured. 

The concentration of activin A was measured using the Human Activin A ELISA Kit, Ref. No. EHACTIVINA, Invitrogen, Carlsbad, CA, USA. In the microplate provided, a target-specific antibody was already immobilized in the wells as a coating. To initiate the process, samples, standards, or controls were added into these wells, where they attached to the capture antibody due to their target specificity. Then the second antibody was added. Following this, a substrate solution was introduced, which reacts with the enzyme-antibody-target complex. This reaction generates a detectable signal that was measured at λ = 450 nm.

The hormonal parameters (including Anti-Müllerian Hormone (AMH), androstenedione (AD), dehydroepiandrosterone sulfate (DHEA-S), FSH, luteinizing hormone (LH), free testosterone (fTest), total testosterone (tTest), and sex-hormone binding globulin (SHBG)) and metabolic measures (lipid profile: cholesterol, high-density lipoprotein (HDL-C), low-density lipoprotein (LDL-C), triglycerides levels, as well as fasting (0′) and post-oral glucose tolerance test (120′) insulin and glucose levels) were measured during hospitalization using methods described in our previous publication [31]. Homeostatic model assessment for insulin resistance (HOMA-IR) was calculated according to the standard formula: HOMA-IR=fasting insulin mUmL×fasting glucose mgdL22.5.

### 2.3. Statistical Analysis

The values were presented as the median along with the 1st quartile, and 3rd quartile. The Shapiro-Wilk test was used to check the normality of the variables, and Levene’s test was employed to assess the homogeneity of variance. When the criteria for normal distribution and equal variance were not met, differences between two groups (non-smoking and smoking women with PCOS) were performed using the non-parametric Mann-Whitney U test, while differences among three subgroups (presented in Table 1) were assessed using Kruskal-Wallis one-way analysis of variance by ranks. Furthermore, a post hoc test was conducted to reveal more specific differences following the Kruskal-Wallis test that were not specified in the initial analysis. Correlation was determined using Spearman’s rank-order correlation coefficient. All analyses were conducted with a significant threshold of *p* < 0.05. To perform statistical analysis, the polish version 13.3 of the Statistica Software Package was used.

## 3. Results

Basic characteristics, including the concentration of investigated parameters in the blood of non-smoking and smoking women with PCOS and non-smoking women without PCOS, are presented in Table 1. No significant differences were detected in the concentration of follistatin and activin A.

We also evaluated the studied parameters in the groups of non-smoking and smoking women with PCOS, additionally divided according to BMI (<25.0 and ≥25.0). These results are summarized in Table 2.

Analyzing the influence of IR on the studied parameters in women with PCOS, both those exposed and not exposed to tobacco smoke, we observed some significant changes, which are presented in Table 3.

Additionally, we also compared the studied parameters between non-smoking and smoking women with PCOS, separately in subgroups with BMI < 25.0 or BMI ≥ 25.0, as well as in subgroups of women with HOMA-IR < 2.0 or HOMA-IR ≥ 2.0. Aside from the significant difference in cotinine concentration, we observed significant changes in activin A levels between non-smoking women with PCOS and BMI ≥ 25.0 and smoking women with BMI ≥ 25.0 (*p* < 0.007), as well as between non-smoking women with PCOS and a HOMA-IR ≥ 2.0 and smoking women with a HOMA-IR ≥ 2.0 (*p* < 0.043). In both cases, activin A concentrations were more than twofold higher in smoking women compared to non-smoking women with PCOS. 

Furthermore, in the group of women with HOMA-IR < 2.0, we found a significant difference in glucose concentration between non-smoking and smoking women with PCOS, while in the subgroup with BMI < 25.0, a significant change was found in AMH concentration between non-smoking and smoking women (*p* < 0.013).

In Table 4, we summarized the correlations between cotinine or follistatin levels and studied hormonal and metabolic parameters. Interestingly, no statistically significant correlations between the concentration of activin A and mentioned in Table 4 parameters were revealed in either the whole group of women with PCOS or in the subgroup of smoking women with PCOS.

**Figure 1:**
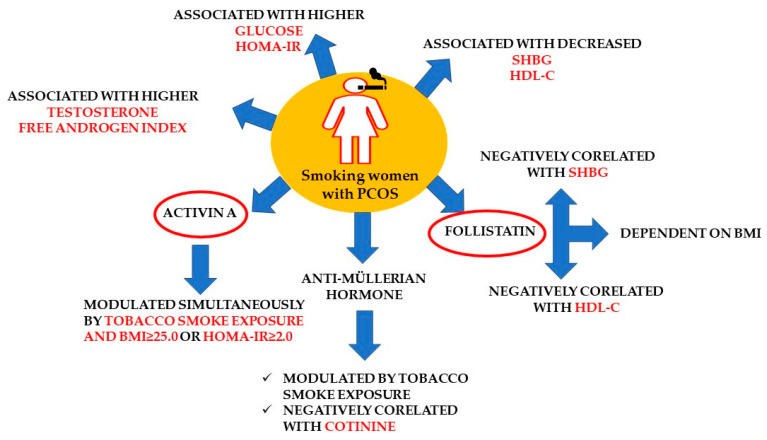
Significant relationships observed in the subgroup of smoking women with PCOS.

Figure 1 summarizes the significant relationships observed in the group of women with PCOS in response to tobacco smoke exposure.

## 4. Discussion

In the present study, we focused on the regulatory peptides follistatin and activin, examining their relationships with selected hormonal and metabolic parameters, with particular emphasis on the potential modulatory effect of tobacco smoke exposure. These peptides, though not routinely assessed, play an important role in maintaining hormonal homeostasis. Given that cigarette smoking is a well-established risk factor for cellular and endocrine dysregulation, we aimed to investigate these interactions in the context of PCOS.

Comparing follistatin levels among non-smoking women with PCOS, smoking women with PCOS, and non-smoking women without PCOS, we did not observe significant differences. To the best of our knowledge, the effect of tobacco smoke exposure on follistatin concentration in women with PCOS has not yet been previously evaluated. However, a meta-analysis by Raeisi et al. [14] summarized follistatin levels in women with and without PCOS, revealing an increased follistatin level in women with PCOS compared to the control group. This meta-analysis included nine publications, with only one study conducted in Europe (Germany). Notably, the German study focused exclusively on pregnant women with or without PCOS [32]. Additionally, Raesi et al. concluded that the elevated concentration of follistatin in women with PCOS was influenced by the age disparity between the PCOS group and the healthy control group. 

Specifically, as the difference in mean age between PCOS patients and healthy controls increased, a notable decrease in the average difference in follistatin levels between the two groups was observed. This finding may explain the lack of significant changes in folistatin concentration in our study, as all participants were of similar age. 

However, when we compared follistatin concentration between the entire group of women with PCOS and the control group, we detected a significant elevation in the concentrations of follistatin in women with PCOS (median: 1377.78 pg/mL; 1st and 3rd quartile: 1211–1622.22 pg/mL) compared to the control group (median: 1166.67 pg/mL; 1st and 3rd quartile: 988.89–1400.00). 

According to Raeisi’s analysis and other studies [14,33], significant associations were found between follistatin levels and obesity. Similarly, we observed a significant increase in follistatin concentration in the blood of women with overweight/obesity (BMI ≥ 25) compared to women with normal weight (BMI < 25.0). Importantly, this change was independent of tobacco smoke exposure. 

Furthermore, follistatin has been identified as a significant marker associated with a higher risk for T2D and IR. Notably, circulating follistatin levels have been proposed as a novel marker of T2D [34,35]. In our study, we initially observed significant differences in follistatin concentration between women with PCOS categorized based on HOMA-IR value (<2.0 and ≥2.0). This finding was consistent in both smoking and non-smoking subgroups with PCOS. Consequently, we examined the relationships between follistatin concentration and glucose levels, insulin levels, and HOMA-IR values. In the overall group of women with PCOS, we found that follistatin concentration exhibited positive correlations with fasting glucose, fasting insulin levels, and HOMA-IR values. Interestingly, these significant relationships were not evident in the subgroup of smoking women with PCOS, which requires further investigation, as it is an interesting observation. 

We also considered the relationship between follistatin and the concentration of FSH or AMH, as these hormones might be modulated by follistatin levels. In both the entire group of women with PCOS and the smoking subgroup of women with PCOS, no significant correlations were found between the follistatin and those hormones. The lack of the significant correlation between follistatin and AMH levels is supported by an experimental study conducted by Kawagishi et al. [36] on an embryonic carcinoma cell line, which demonstrated that the maximum bioactivity of recombinant human AMH was not altered by follistatin. 

We found significant differences in AMH concentrations between smoking and non-smoking women with PCOS and the control group. Furthermore, post hoc analysis revealed significant differences in AMH concentrations between smoking women with PCOS and the control group, as well as between non-smoking women with PCOS and the control group. However, no significant differences were observed in AMH concentrations when comparing non-smoking and smoking women with PCOS (*p* = 0.253). Additionally, in the entire group of women with PCOS, AMH levels were negatively associated with cotinine concentration. The effect of tobacco smoke exposure on AMH concentration is generally inconclusive. Some studies indicate that exposure to tobacco smoke does not significantly affect AMH concentration or reduce ovarian reserve [37,38], while other authors report that smoking decreases AMH concentration [38,39]. A study by Plante et al. [39] on late-reproductive and perimenopausal women showed that exposure to tobacco smoke was associated with lower AMH concentrations, which was attributed to the depletion of antral follicles. These findings may explain the lower AMH concentration in the group of smoking women with PCOS compared to non-smoking women with PCOS, as well as the negative correlation between cotinine and AMH concentration. However, due to the small number of cases, these results should be considered preliminary and warrant further investigation.

We hypothesized that determining follistatin concentration in the blood of women with PCOS could be a useful marker indicating fertility and might be directly associated with AMH concentration. Follistatin has even been suggested as a possible marker or therapeutic target in PCOS [14]. Unfortunately, based on our current results, we could not confirm this hypothesis. It is possible that more studies with larger sample sizes are needed to observe these relationships. Therefore, the present study should be considered preliminary. 

Few alterations were detected when evaluating activin A concentration in the blood of women with PCOS. Our results suggest that PCOS alone does not influence its concentration, while BMI and HOMA-IR could be considered crucial factors affecting activin A levels. We observed a greater than two-fold increase in activin A concentration among smoking women with PCOS and a BMI ≥ 25.0 compared to their non-smoking counterparts with similar BMI ≥ 25.0. A comparable elevation was noted when comparing smoking and non-smoking women with PCOS and HOMA-IR ≥ 2.0. Similarly, activin A concentrations were two-fold higher in subgroups with elevated BMI (≥25.0) or HOMA-IR (≥2.0) than in those with lower BMI (<25.0) or HOMA-IR (<2.0), respectively. These findings suggest that insulin resistance and overweight/obesity may influence activin A levels. Additionally, tobacco smoke exposure in women with PCOS, particularly in the presence of insulin resistance or abnormal weight, appears to modulate activin A concentrations. Therefore, we hypothesize that in women with PCOS, smoking, in conjunction with insulin resistance and/or abnormal weight, may influence activin A levels.

The relationship between HOMA-IR and activin A was already observed in the study conducted on a prediabetic population [40,41], suggesting that activin A may be useful in assessing IR. Since IR and T2D are common complications in PCOS, associations between activin A concentration and glucose metabolism parameters should be evaluated more thoroughly. Our study supports these observations; however, when we analyzed the correlations between activin A concentrations and hormonal or metabolic parameters, no significant relationships were observed, either in the entire group of women with PCOS or in the smoking subgroup.

Considering hormonal and metabolic parameters in the context of tobacco smoke exposure, we observed significant changes in the concentration of SHBG, fasting glucose, insulin, HDL-C, and the values of HOMA-IR and FAI among these groups (Table 1). Additionally, in the entire group of women with PCOS, we revealed a negative correlation between cotinine concentration and SHBG levels, while in the subgroup of smoking women with PCOS, we found a positive correlation between cotinine concentration and both fTest or tTest and AD. These results align with the findings of Ö. E. Özay and A. C. Özay [17], who also observed a positive correlation between tTest (r = 0.448, *p* < 0.001) or fTest (r = 0.423, *p* < 0.001) with the number of cigarettes smoked per day, while SHBG concentration showed a negative correlation (r = −0.454, *p* < 0.001) with the number of cigarettes smoked per day. 

In our previous study involving 606 Caucasian women, we distinctly demonstrated that the most significant alterations in the blood of women with PCOS were linked to the concentrations of SHBG, HDL-C, triglycerides, and the FAI value [42]. In the study conducted by Pau et al. [43], exposure to tobacco smoke in PCOS was associated with elevated triglyceride concentration, even when adjusted for BMI. Moreover, Rehan et al. [44] confirmed that nicotine influences endocrine homeostasis, leading to imbalances in gonadal steroid hormones and adrenal corticosteroid hormones. The authors also noted that nicotine may potentially interfere with the binding of estradiol, progesterone, cortisol, and testosterone, disrupting their transport and homeostasis in the bloodstream [44]. 

In summary, activin A and follistatin concentrations appear to be more affected by disruptions in glucose metabolism and BMI than by tobacco smoke exposure. This is evidenced by the lack of significant correlations between cotinine levels and these protein concentrations in both the overall group and the subgroup of smoking women with PCOS.

Nevertheless, the significant alterations in specific metabolic and hormonal parameters observed with tobacco smoke exposure suggest that smoking is an additional factor that adversely impacts homeostasis in women with PCOS. We hope that our study highlights trends that could enhance understanding of key metabolic and hormonal changes in PCOS, particularly in identifying new useful parameters involved in the development, progression, and differentiation of this disease.

However, several limitations should be considered when interpreting these study results. The relatively small sample size of both PCOS patients and the control group, the variability in tobacco smoke exposure (with participants reporting smoking between 2 and 27 cigarettes per day and cotinine concentrations ranging from 15.65 to 69.94 ng/mL), and the inclusion of control group participants who, despite having normal hormonal levels and being excluded from PCOS diagnosis, exhibited some menstrual disorders, all suggest these findings should be regarded as preliminary.

## Figures and Tables

**Table 1:** Basic characteristics of women with PCOS and the control group, as well as the level of selected biochemical parameters with particular emphasis on the concentration of follistatin and activin A.

Parameters	Women			
	Smoking with PCOS	Non-Smoking with PCOS	Non-Smoking without PCOS	*p* Value between 3 Subgroups
	n = 23	n = 37	n = 28	
Age (years)	26.46 ± 4.47 25.50 (23.00–29.00)	24.51 ± 4.79 24.50 (22.00–27.00)	26.71 ± 5.87 27.00 (22.00–31.00)	0.324
BMI (kg/m^2^)	27.30 ± 7.57 25.55 (21.01–31.25)	25.15 ± 7.14 23.66 (20.42–29.49)	22.62 ± 4.58 21.63 (20.57–23.03)	0.064
FG	5.48 ± 3.93 6.00 (2.00–8.00)	6.02 ± 5.23 5.00 (1.50–9.00)	NA	NA
Cotinine (ng/mL)	44.72 ± 18.29 */** 78.87 (30.75–60.42)	0.50 ± 0.34 0.41 (0.00–0.41)	0.46 ± 0.14 0.41 (0.00–0.41)	0.000
LH (lU/L)	9.28 ± 5.02 7.00 (5.71–10.50)	8.71 ± 5.38 7.48 (5.53–9.80)	6.09 ± 4.57 5.30 (2.73–8.59)	0.057
FSH (lU/L)	6.24 ± 1.24 6.18 (5.35–6.67)	5.95 ± 1.64 5.86 (5.31–7.11)	6.73 ± 2.11 6.84 (5.84–8.55)	0.105
DHEA-S (µg/mL)	306.14 ± 161.93 252.00 (192.00–362.00)	326.50 ± 127.11 301.00 (223.00–397.00)	252.51 ± 143.42 230.00 (164.00–295.00)	0.059
SHBG (nmol/L)	49.56 ± 34.26 * 39.95 (28.40–55.50)	62.85 ± 43.64 58.55 (34.40–74.70)	68.91 ± 37.07 62.50 (50.20–71.60)	0.034
tTest (ng/mL)	0.39 ± 0.17 * 0.35 (0.29–0.57)	0.41 ± 0.17 0.38 (0.31–0.51)	0.30 ± 0.22 0.26 (0.16–0.32)	0.008
fTest (pg/mL)	3.26 ± 3.18 1.98 (1.47–4.11)	3.14 ± 2.58 2.18 (1.51–3.88)	2.22 ± 2.25 1.45 (0.71–2.06)	0.100
AD (ng/mL)	2.11 ± 0.60 2.13 (1.65–2.57)	2.66 ± 1.04 * 2.48 (1.90–3.39)	2.06 ± 0.73 1.86 (1.59–2.20)	0.010
FAI	4.28 ± 3.57 * 3.48 (1.54–5.67)	3.55 ± 3.12 2.73 (1.74–3.95)	1.83 ± 1.54 1.2 (0.72–2.61	0.005
AMH (ng/mL)	5.12 ± 2.64 * 5.20 (3.19–6.39)	6.95 ± 3.53 * 6.66 (3.98–9.34)	3.18 ± 2.18 2.47 (1.63–4.49)	0.000
Follistatin (pg/mL)	1408.59 ± 293.15 1333.00 (1211.11–1588.89)	1419.68 ± 455.43 1366.67 (1133.33–1622.22)	1392.06 ± 426.79 1300.06 (1066.67–1522.22)	0.850
Activin A (pg/mL)	333.77 ± 350.03 189.00 (116.50–486.50)	748.58 ± 1162.39 289.00 (141.50–609.00)	452.69 ± 501.83 301.50 (146.50–486.50)	0.408
Cholesterol (mg/dL)	166.41 ± 27.87 164.50 (148.00–188.00)	167.09 ± 39.46 165.00 (144.00–195.00)	171.43 ± 30.55 163.00 (146.00–198.00)	0.976
HDL-C (mg/dL)	54.08 ± 14.04 * 52.50 (45.90–66.50)	56.49 ± 15.07 57.40 (49.70–63.70)	67.84 ± 15.28 68.80 (58.00–75.20)	0.006
LDL-C (mg/dL)	89.39 ± 24.78 82.34 (72.50–108.90)	91.36 ± 28.14 92.37 (72.80–114.76)	89.71 ± 22.57 83.32 (74.40–106.52)	0.790
Triglycerides (mg/dL)	115.16 ± 65.81 89.80 (73.80–137.00)	100.15 ± 68.78 73.65 (57.20–118.00)	75.81 ± 29.99 66.50 (53.70–86.70)	0.052
Glucose 0′ (mg/dL)	87.96 ± 7.96 * 86.65 (81.00–92.00)	81.60 ± 13.23 83.60 (79.50–87.50)	81.65 ± 5.19 80.50 (78.50–86.80)	0.028
Glucose 120′(mg/dL)	118.41 ± 41.95 111.00 (95.50–133.00)	106.67 ± 33.41 96.65 (85.40–125.00)	94.40 ± 27.06 93.50 (80.20–103.00)	0.085
Insulin 0′ (mU/mL)	9.55 ± 7.76 7.52 (5.37–11.40)	9.72 ± 7.00 6.97 (4.98–12.50)	5.34 ± 2.52 5.93 (3.49–6.77)	0.020
HOMA-IR	2.17 ± 2.11 * 1.54 (1.09–2.41)	2.07 ± 1.60 * 1.50 (0.98–2.76)	1.09 ± 0.52 1.18 (0.70–1.41)	0.021

* *p* < 0.05 when compared to non-smoking women without PCOS; ** *p* < 0.05 when compared to non-smoking women with PCOS. NA—not applicable.

**Table 2:** The effect of tobacco smoking and BMI on examined parameters in PCOS women.

Variables	Non-Smoking Women with PCOS		Smoking Women with PCOS	
	BMI < 25.0	BMI ≥ 25.0	BMI < 25.0	BMI ≥ 25.0
	n = 23	n = 15	n = 10	n = 12
Age (years)	24.17 ± 3.21 24.00 (21.00–27.00)	26.31 ± 3.16 26.00 (26.00–29.00)	27.40 ± 5.65 28.50 (21.00–31.00)	25.67 ± 3.37 24.00 (23.50–27.00)
BMI (kg/m^2^)	21.24 ± 1.81 20.86 (20.26–24.49)	31.81 ± 4.53 * 31.23 (27.86–34.37)	20.63 ± 2.23 20.88 (19.96–22.04)	32.86 ± 5.58 ** 30.87 (29.22–37.91)
FG	5.79 ± 4.92 5.00 (1.00–9.00)	5.8 ± 5.31 5.00 (1.00–9.50)	5.90 ± 4.27 5.50 (3.00–7.00)	5.75 ± 3.31 6.00 (2.50–8.50)
Cotinine (ng/mL)	0.49 ± 0.32 0.41 (0.00–0.41)	0.44 ± 0.12 0.41 (0.00–0.41)	40.26 ± 20.06 41.62 (19.42–57.01)	48.46 ± 16.65 56.75 (33.05–63.27)
LH (lU/L)	9.81 ± 5.36 8.92 (6.52–11.15)	6.68 ± 1.91 * 6.53 (5.46–7.88)	8.09 ± 3.59 6.99 (5.75–10.00)	10.28 ± 5.93 7.47 (5.67–15.05)
FSH (lU/L)	6.32 ± 1.30 5.94 (5.48–7.55)	5.61 ± 1.37 5.39 (5.02–6.26)	6.40 ± 1.24 6.36 (5.71–6.77)	6.11 ± 1.28 5.77 (5.27–6.79)
DHEA-S (µg/mL)	302.50 ± 111.82 274.50 (222.00–365.50)	361.63 ± 119.45 304.00 (287.00–443.00)	249.20 ± 66.26 252.00 (192.00–295.00)	353.58 ± 202.65 284.50 (199.50–459.50)
SHBG (nmol/L)	72.92 ± 38.63 69.00 (56.20–82.35)	38.78 ± 16.61 * 36.9 (26.00–46.95)	72.29 ± 38.28 63.40 (45.70–80.70)	30.46 ± 13.35 ** 29.75 (18.35–38.90)
tTest (ng/mL)	0.39 ± 0.16 0.35 (0.27–0.47)	0.46 ± 0.14 0.40 (0.34–0.55)	0.30 ± 0.09 0.34 (0.27–0.36)	0.47 ± 0.18 ** 0.53 (0.30–0.63)
fTest (pg/mL)	2.49 ± 1.77 1.83 (1.20–3.50)	4.00 ± 3.23 3.22 (1.80–4.77)	1.71 ± 0.85 1.52 (1.45–1.93)	4.55 ± 3.84 ** 3.80 (1.96–5.73)
AD (ng/mL)	2.76 ± 0.92 2.54 (1.96–3.41)	2.57 ± 1.03 2.35 (1.95–2.79)	2.09 ± 0.56 2.22 (1.73–2.45)	2.13 ± 0.65 2.03 (1.61–2.78)
FAI	2.20 ± 1.22 2.00 (1.18–2.82)	5.08 ± 3.54* 3.75 (2.77–7.24)	1.89 ± 1.24 1.53 (1.07–2.49)	6.27 ± 3.68 ** 5.49 (3.60–7.91)
AMH (ng/mL)	7.48 ± 3.21 6.78 (5.65–9.86)	6.25 ± 2.79 6.03 (3.87–8.04)	5.10 ± 3.31 5.00 (1.41–6.23)	5.13 ± 2.00 5.50 (3.40–6.75)
Follistatin (pg/mL)	1257.87 ± 243.15 1200.00 (1105.56–1461.11)	1747.22 ± 496.56 * 1605.57 (1422.22–1772.22)	1267.78 ± 170.72 1233.33 (1166.67–1377.78)	1525.93 ± 327.37 ** 1494.44 (1294.44–1722.22)
Activin A (pg/mL)	496.29 ± 629.07 209.00 (131.50–609.07)	1186.50 ± 1529.89 * 336.50 (229.00–1654.00)	365.00 ± 227.36 364.00 (121.50–536.50)	307.753 ± 435.86 171.50 (106.50–206.50)
Cholesterol (mg/dL)	168.54 ± 30.20 165.00 (149.00–190.50)	175.88 ± 35.56 175.00 (147.00–197.00)	162.70 ± 21.50 159.50 (148.00–186.00)	169.50 ± 32.88 168.00 (147.50–193.00)
HDL-C (mg/dL)	62.63 ± 10.23 61.85 (56.00–66.10)	50.25 ± 10.76 * 50.10 (41.20–60.05)	63.89 ± 10.02 66.85 (52.90–69.90)	45.91 ± 11.56 ** 47.40 (34.30–55.25)
LDL-C (mg/dL)	91.12 ± 24.79 92.53 (73.27–105.40)	99.33 ± 26.27 96.79 (77.05–119.74)	83.28 ± 18.60 78.98 (72.42–103.00)	94.48 ± 28.76 90.05 (72.60–114.35)
Triglycerides (mg/dL)	74.02 ± 28.90 64.40 (53.45–80.75)	131.46 ± 76.50 * 105.50 (76.50–156.00)	78.67 ± 26.97 72.15 (54.50–102.00)	145.57 ± 73.85 ** 114.50 (86.10–209.00)
Glucose 0′ (mg/dL)	83.43 ± 5.57 83.45 (79.65–86.75)	85.34 ± 5.83 86.10 (80.85–90.20)	85.14 ± 5.09 86.250 (80.90–87.60)	90.31 ± 9.29 88.50 (82.25–96.85)
Glucose 120′ (mg/dL)	100.61 ± 22.87 95.40 (84.75–113.50)	122.83 ± 38.47 118.00 (90.85–148.50)	110.06 ± 21.53 111.00 (95.50–124.00)	125.36 ± 53.52 111.00 (91.65–137.00)
Insulin 0′(mU/mL)	6.44 ± 2.56 6.11 (4.33–7.29)	14.40 ± 7.14 * 12.85 (9.66–18.50)	5.66 ± 2.92 5.02 (3.52–7.38)	12.71 ± 9.10 ** 10.65 (7.11–14.75)
HOMA-IR	1.35 ± 0.62 1.26 (0.85–1.56)	3.07 ± 1.63 * 2.76 (2.00–3.85)	1.18 ± 0.58 1.09 (0.81–1.50)	2.99 ± 2.56 ** 2.35 (1.55–3.26)

* *p* < 0.05 when compared to non-smoking subgroup with BMI < 25.00; ** *p* < 0.05 when compared to smoking subgroup with BMI < 25.00.

**Table 3:** The effect of tobacco smoking and HOMA-IR value on examined parameters in PCOS women.

Variables	Non-Smoking Women with PCOS		Smoking Women with PCOS	
	HOMA-IR < 2.0	HOMA-IR ≥ 2.0	HOMA-IR < 2.0	HOMA-IR ≥ 2.0
	n = 25	n = 13	n = 14	n = 8
Age (years)	24.12 ± 3.32 24.00 (22.00–26.00)	26.54 ± 2.88 * 27.00 (25.00–29.00)	27.70 ± 5.69 27.50 (23.00–34.00)	25.75 ± 2.60 25.50 (24.00–28.00)
BMI (kg/m^2^)	22.14 ± 2.74 21.10 (20.34–23.94)	32.20 ± 5.42 * 32.83 (29.49–34.45)	23.30 ± 4.95 21.57 (19.96–25.95)	34.32 ± 6.17 ** 34.22 (30.48–39.56)
FG	5.31 ± 4.98 4.00 (1.00–9.00)	7.39 ± 5.01 8.00 (3.00–10.00)	5.71 ± 4.12 6.00 (3.00–7.00)	6.00 ± 3.78 6.00 (2.50–9.50)
Cotinine (ng/mL)	0.49 ± 0.32 0.41 (0.–0.41)	0.45 ± 0.13 0.41 (0.00–0.41)	40.64 ± 18.50 41.62 (24.00–56.75)	50.47 ± 18.64 61.22 (31.40–64.62)
LH (lU/L)	8.72 ± 5.26 7.44 (5.73–9.80)	8.04 ± 3.10 7.51 (6.35–9.51)	8.21 ± 3.82 6.82 (5.71–10.00)	11.16 ± 6.50 8.48 (5.74–16.65)
FSH (lU/L)	6.23 ± 1.60 5.99 (5.49–7.57)	5.80 ± 0.78 * 5.46 (5.32–6.20)	6.44 ± 1.19 6.54 (6.13–6.96)	5.89 ± 1.31 5.66 (5.27–5.78)
DHEA-S (µg/mL)	320.92 ± 122.40 281.00 (222.00–405.00)	344.62 ± 116.35 304.00 (293.00–365.00)	298.79 ± 114.98 276.50 (226.00–362.00)	319.00 ± 231.98 213.50 (183.50–367.50)
SHBG (nmol/L)	69.80 ± 39.20 67.80 (45.30–77.60)	39.77 ± 19.53 * 34.40 (25.20–51.60)	62.65 ± 36.27 53.45 (29.90–80.20)	26.41 ± 11.36 ** 26.40 (15.55–34.45)
tTest (ng/mL)	0.36 ± 0.13 0.33 (0.27–0.46)	0.51 ± 0.17 * 0.45 (0.40–0.65)	0.37 ± 0.16 0.34 (0.29–0.39)	0.44 ± 0.19 0.43 (0.28–0.63)
fTest (pg/mL)	2.34 ± 1.63 2.02 (1.20–2.72)	4.76 ± 3.29 * 3.93 (3.04–5.47)	2.80 ± 3.54 1.60 (1.45–2.25)	4.07 ± 2.43 3.91 (2.57–5.60)
AD (ng/mL)	2.59 ± 0.89 2.37 (1.91–2.85)	2.88 ± 1.13 2.50 (2.01–3.79)	2.00 ± 0.59 2.13 (1.33–2.34)	2.31 ± 0.60 2.29 (1.79–2.78)
FAI	2.28 ± 1.36 1.98 (1.19–2.80)	5.52 ± 3.71 * 3.95 (3.33–8.71)	2.89 ± 2.41 2.08 (1.43–3.80)	6.71 ± 4.10 ** 5.49 (4.41–8.61)
AMH (ng/mL)	7.20 ± 3.41 6.96 (3.98–9.71)	6.48 ± 2.59 * 6.918 (5.16–7.39)	5.56 ± 2.78 5.46 (3.61–7.10)	5.00 ± 2.48 5.50 (2.49–6.18)
Follistatin (pg/mL)	1268.00 ± 243.625 1244.44 (1111.11–1455.56)	1734.19 ± 440.15 * 1622.22 (1555.56–1722.22–)	1315.08 ± 218.19 1277.78 (1211.11–1455.56)	1572.22 ± 5348.29 1494.44 (1294.44–1872.22)
Activin A (pg/mL)	484.70 ± 714.34 211.50 (136.50–386.50)	1332.27 ± 1573.17 * 316.50 (281.50–19571.50)	330.43 ± 235.23 211.50 (146.50–536.50)	339.63 ± 514.52 146.50 (86.50–321.50)
cholesterol (mg/dL)	160.80 ± 27.48 155.50 (144.00–174.00)	187.08 ± 34.35 195.00 (157.00–201.00)	163.43 ± 28.97 164.50 (149.00–186.00)	171.63 ± 26.87 170.00 (147.00–193.00)
HDL-C (mg/dL)	61.16 ± 11.24 60.70 (53.30–64.50)	50.00 ± 10.93 * 50.90 (41.10–58.10)	60.75 ± 11.32 62.55 (52.10–67.60)	42.41 ± 10.41 ** 45.30 (31.55–48.60)
LDL-C (mg/dL)	85.86 ± 22.72 83.90 (67.20–101.58)	106.78 ± 25.00 * 116.20 (88.94–123.34)	85.40 ± 21.85 82.34 (72.42–103.00)	96.37 ± 29.47 93.30 (72.60–114.35)
Triglycerides (mg/dL)	68.94 ± 22.14 63.00 (52.30–74.70)	151.52 ± 75.27 * 142.00 (92.60–159.00)	87.12 ± 41.46 79.80 (62.90–90.20)	164.23 ± 74.00 ** 145.00 (101.00–232.00)
Glucose 0′ (mg/dL)	81.78 ± 4.80 82.30 (77.60–84.40)	87.59 ± 4.80 * 88.90 (83.70–91.00)	85.19 ± 4.64 86.25 (81.00–87.60)	92.80 ± 10.38 92.85 (83.30–100.00)
Glucose 120′ (mg/dL)	99.85 ± 24.57 92.90 (83.50–115.00)	123.47 ± 34.17 * 108.00 (95.90–148.00)	105.90 ± 23.12 107.50 (77.60–124.00)	140.23 ± 58.54 125.00 (105.50–154.50)
Insulin 0′ (mU/mL)	5.90 ± 1.76 6.08 (4.38–6.89)	16.36 ± 6.44 * 13.40 (12.50–18.70)	5.69 ± 2.07 5.79 (4.10–7.38)	16.31 ± 9.53 ** 12.75 (11.30–16.60)
HOMA-IR	1.20 ± 0.38 1.17 (0.86–1.48)	3.53 ± 1.46 * 2.91 (2.76–3.87)	1.19 ± 0.43 1.22 (0.83–1.50)	3.87 ± 2.79 ** 2.95 (2.41–3.68)

* *p* < 0.05 when compared to non-smoking subgroup with HOMA-IR<2.00; ** *p* < 0.05 when compared to smoking subgroup with HOMA-IR < 2.00.

**Table 4:** The correlation coefficients between the concentration of cotinine or follistatin and the value of other analyzed parameters in the whole group of women with PCOS and in the smoking group of women with PCOS.

Variable	Whole Group of Women with PCOS		Smoking Women with PCOS	
	Cotinine (ng/mL)	Follistatin (pg/mL)	Cotinine (ng/mL)	Follistatin (pg/mL)
LH (lU/L)	NS	NS	NS	NS
FSH (lU/L)	NS	NS	NS	NS
SHBG (nmol/L)	r = −0.35; *p* < 0.007	r = −0.32; *p* < 0.014	NS	r = −0.48; *p* = 0.025
DHEA-S (µg/mL)	NS	NS	NS	NS
tTest (ng/mL)	NS	NS	r = 0.63; *p* < 0.001	NS
fTest (pg/mL)	NS	NS	r = 0.52; *p* < 0.012	NS
AD (ng/mL)	NS	NS	r = 0.50; *p* < 0.018	NS
FAI	NS	r = 0.33; *p* < 0.012	NS	NS
AMH (ng/mL)	r = −0.30; *p* < 0.023	NS	NS	NS
Follistatin (pg/mL)	NS	NS	NS	NS
Activin A (pg/mL)	NS	NS	NS	NS
Cholesterol (mg/dL)	NS	r = 0.30; *p* < 0.021	NS	NS
HDL-C (mg/dL)	NS	r = −0.31; *p* < 0.015	NS	r = −0.55; *p* < 0.008
LDL-C (mg/dL)	NS	r = 0.310; *p* < 0.016	NS	NS
Triglycerides (mg/dL)	NS	r = 0.47; *p* < 0.000	NS	NS
Glucose 0′ (mg/dL)	r = 0.26; *p* < 0.049	r = 0.33; *p* < 0.010	NS	NS
Glucose 120′ (mg/dL)	NS	NS	NS	NS
Insulin 0′ (mU/mL)	NS	r = 0.45; *p* < 0.000	NS	NS
HOMA-IR	NS	r = 0.47; *p* < 0.000	NS	NS

## Data Availability

The data presented in this study are available upon request from the corresponding author.
